# Use of traditional medicine for dental care by different ethnic groups in New Zealand

**DOI:** 10.1186/s12903-020-01272-7

**Published:** 2020-10-12

**Authors:** Jun Guo, Kah Seng Low, Li Mei, Jia Hui Li, Wenwen Qu, Guangzhao Guan

**Affiliations:** 1 Department of Stomatology, Sichuan Academy of Medical Sciences and Sichuan Provincial People’s Hospital, 32# West Section 2, 1st Ring Road, Chengdu, 610072 China; 2 Department of Oral Sciences, Sir John Walsh Research Institute, Faculty of Dentistry, University of Otago, Dunedin, New Zealand; 3 School of Pharmacy, University of Otago, Dunedin, New Zealand; 4 Department of Gynaecology, West China Second University Hospital, Sichuan University, Chengdu, 610041 China; 5 Department of Oral Diagnostic and Surgical Sciences, Faculty of Dentistry, University of Otago, 310 Great King Street, Dunedin, 9016 New Zealand

**Keywords:** Traditional medicine, Alternative medicine, Dentistry

## Abstract

**Background:**

There is an increasing public interest in the use of TM internationally, yet there is a paucity of research on the use of TM by the public in the dental setting. This study aimed to explore the views, use of and access to TM in dentistry among different ethnic groups residing in New Zealand.

**Methods:**

Qualitative study and in-depth interviews were used. An individual semi-structured questionnaire was used to collect data. Interviews were recorded, transcribed, and analysed using an inductive approach to identify the main themes.

**Results:**

Three main themes were extracted from interviews with 14 participants from diverse cultural backgrounds: [1] the perspectives of TM varied among different ethnic groups and included the involvement of spirituality, the environment, knowledge and usage of TM. [2] The TM that was used by different ethnic groups included plants, herbs, massage, and other forms of healing. Reasons for choosing traditional or western medicines generally included family tradition, access to TM, and finding a competent traditional healer. [3] The barriers in accessing TM included the paucity of traditional healers, difficulty accessing plants and cost, therefore most would look for a substitution or alternative treatment.

**Conclusion:**

Even though the access to these TM in New Zealand was a challenge for the majority of the participants, they are still considered the first-line treatment for the majority. This study provided dental practitioners an insight into the different sort of TM used by the population. By understanding and acknowledging the use of TM, dental practitioners could create a supportive environment for patients to disclose their use of TM and allow them to educate patients on the use of TM.

## Background

Since ancient times, humans sought out cures for disease in nature. This was known as traditional medicine (TM), and it was considered the oldest form of healthcare [1]. It has been used over many centuries by diverse ethnic groups, and invaluable traditional knowledge has accumulated over many generations [2]. The history of TM can be traced from an ancient civilization, and it was evident that this information was passed down to the next generation as they played an important role as the primary source of health care [2].

With the introduction of western medicine, the medical systems based on traditional oriental medicine become less mainstream [3]. However, TM came before modern medicine and was used to treat a variety of medical conditions [1]. According to the World Health Organisation, 80% of the world’s people depended on TM (herbal) for their primary healthcare needs as these plant extracts were readily accessible, affordable and culturally appropriate [4]. Also, the economic benefits could be achieved through the development of indigenous medicines and the use of herbal medicines to treat various diseases [5]. Around 25% of the medical drugs were based on herbs and their derivatives in developed countries [6].

TM was originally used to treat medical conditions, however, there was an emerging trend of TM being used in dentistry to relieve tooth pain, periodontal inflammation and oral mucosal diseases [2]. A study reported that traditional healers in India used 2500 plant species and that 100 species of plants served as regular sources of medicine [2]. Ayurveda, Yoga, Unani, Siddha and Homeopathy, an ancient India traditional medical discipline, has found acceptance in the management of oral diseases. It treats a patient as a whole not as a group of individual parts. It is aimed at healing the body, mind and soul. The important roles of Ayurveda, Unani and Homeopathy systems in the management of oral diseases have been already published in several articles [7–9]. Indigenous people used natural toothbrushes which were made from healing plants to brush their teeth [2]. It was suggested that the healing plants contain volatile oils which stimulate blood circulation, tannins that tighten and cleanse gum tissue and other components, such as vitamin C to maintain healthy gums [2]. In Mexico, dental services were difficult to access in the urban and rural areas due to the cost, and consequently, it limited people from accessing the appropriate drugs [10, 11]. For these reasons, herbal remedies were used in Mexico, despite the lack of scientific support for their use, dosage and effects [12]. Another study conducted in the United States showed that those that could not afford to visit dentists might use alternative sources of care for toothache pain relief [13]. These could include home remedies or over the counter and prescription medications [13]. However, due to the lack of a randomised controlled clinical trial, the toxicity of these herbal medicines was not known, and only a few plants have been approved for their commendable medicinal properties [2]. Most patients believed herbal medication to be benign and not cause any severe toxicity, and a number of patients use them without caution because these plants come from natural sources. However, despite the general belief, herbal medicines could cause severe toxicity and even death if used without caution [2]. Therefore, it is important for dental practitioners to study and understand the traditional medicinal plants being used in the New Zealand population. Furthermore, dental practitioners need to be informed regarding the use, safety and effectiveness of the various traditional medicines and over-the-counter products. By incorporating this knowledge into the dental education model core curriculum, this would allow dental practitioners to integrate their professional dental treatment with TM to provide the optimal treatment for their patients. As a result, the best possible outcomes could be achieved. In addition, the crux of understanding the interactions of plant extracts with the body and other medications would allow dental practitioners to conduct appropriate treatments, as many of these extracts have anti-inflammatory properties and prevent bleeding, which is important in dental treatment [14]. The following properties that were derived from plants were of widespread interest in dentistry; these include: antiseptics, antibacterial, antimicrobial, antifungal, antioxidant, antiviral and analgesic [15]. For example, several plant extracts such as propolis, noni fruit, burdock root, and neem leaf had been used in the field of periodontics and endodontics as intra-canal medications with excellent results [16]. This showed that herbal agents could be the next novel medicine in the global dental therapy. However, there are still significant gaps in the research.

There is an increasing public interest in the use of TM internationally, yet there is a paucity of research on the use of TM by the public in the dental setting. Hence, the study aimed to explore the views, use of and access to TM in dentistry among different ethnic groups residing in New Zealand.

## Methods

This was a qualitative study conducted between 10/2018–09/2019. This study was approved by the Human Research Ethics Committee University of Otago (D18/336)**.** The sample size was based on previous studies [17–19] and recruitment was stopped once thematic saturation was reached. Participants were recruited purposively at three major cities in New Zealand, which had a high proportion of people that have the knowledge or used TM in the field of dentistry. The inclusion criteria were community elders, leaders and adult members that have had experience or knowledge of TM. Participants must have at least conversational English; non-English speakers could be interviewed if a translator was available. The exclusion criteria were children and adults who have limited knowledge about TM or do not use TM.

All interviews were semi-structured, and followed a piloted interview guide at either their homes or at the local library at Auckland, Hamilton and Dunedin. Following informed consent, the semi-structured interviews were facilitated in person using a pre-determined question set (Table 1). These questions were designed and adjusted by reviewing previous research so that they could be applied in this study. The questions were structured with open-ended questions to allow participants to elaborate. Socio-demographic details (age and ethnicity) were also collected at the time of the interview.

**Table 1:** Interview Questionnaire

1. **What is your experience of TM in New Zealand** What do you consider to be TM? What do you know about the TM?	
2. **Do you use any TM for dental/non-dental pathologies affecting the orofacial region? This can be from orofacial pain to oral mucosal diseases** If so what are they and what do they treat? How long do you use it for?	
3. **TM can have lots of values and significance for people for different reasons. What are some of the reasons you use TM?** Why not? What situation is TM used in? what situations aren’t	
4. **What are the ways that you access the TM?** How does this differ to when in NZ vs your home country? What barriers exist to accessing TM? How has access to TM changed over time? Were there any changes in the use of TM after immigrating to New Zealand?	
5. **How do people in your community view traditional medicine**? How have others responded to your use of TM? What is your experience of the health care professionals and TM? How has this changed the way you use traditional or NZ medical systems?	

*TM* Traditional medicine

The interviews were audio-recorded and converted to a transcript by the investigators (KL and GZG). A database of interview transcripts and field notes from observations was created, stored, and handled using the NVivo 12 (QSR International) computer program. These transcriptions were reviewed by the participants for accuracy. The transcriptions were analysed using a thematic approach to identify patterns to obtain key themes within the qualitative data. The main author collected the qualitative data and identified keywords or phrases in the data that were mentioned numerous times by different participants. The analysis consisted of reading transcripts and field notes to identify recurrent concepts, which were coded according to the topic. The codes were grouped into similar categories, subcategories and described in detail. Each category was assessed, and common themes were then identified. A basic content analysis of the transcripts that measured the frequency of words spoken by participants was used to create word clouds for each major theme.

As described by Morse et al., the validity, reliability, and generalizability were used as a framework to ensure the rigour in this study [20]. This was achieved by the purposive sampling, the iterative process of data collection and analysis, following up on new and recurrent concepts, and tracking and checking the changes.

## Results

The 14 participants interviewed were from different ethnic groups (Chinese, Korean, Pacific and Maori). These participants were given a numerical code from P1 to P14 to indicate how many participants were using this data (Table 2). The fourteen participants that took part in this study had some understandings and used TM in relation to dental care. A Chinese translator was required for the Chinese ethnicity, and the rest of the ethnicities could converse in English fluently. Eight participants were female and six were male. The mean age of the participants was 49 ± 11 years old. From the study, three main themes along with several sub-themes associated with them were postulated (Table 3). The first theme concentrated on the different perspective of each participant had on TM; the second theme outlined the current practice and experience of the participants; the third theme focused on barriers to accessing TM in New Zealand.

**Table 2:** Demographics of the participants

		N (%)
**Gender**	Male	6 (43%)
	Female	8 (57%)
**Age (years)**	30–39	4 (29%)
	40–49	1 (7%)
	50–59	6 (43%)
	> 60	3 (21%)
**Ethnicity**	Chinese	5 (36%)
	Pacific Islanders	4 (29%)
	Maori	3 (21%)
	European	2 (14%)

**Table 3:** Themes and sub-themes

1. Perspectives of TM a. Different definition of TM b. Involvement of spirituality c. Involvement with the environment d. Knowledge and usage of TM	
2. Current practice and experience with TM a. TM practices in dental/non dental pathologies in New Zealand b. Relationship between traditional and western Medicine	
3. Barriers to accessing TM in New Zealand a. Lack of Traditional Healers b. Lack of Traditional Plants c. Cost of TM d. Adaptation and substitution	

*TM* Traditional medicine

### Theme 1: perspectives of TM

#### Sub-theme 1a: different definition of TM

**Fig. 1:**
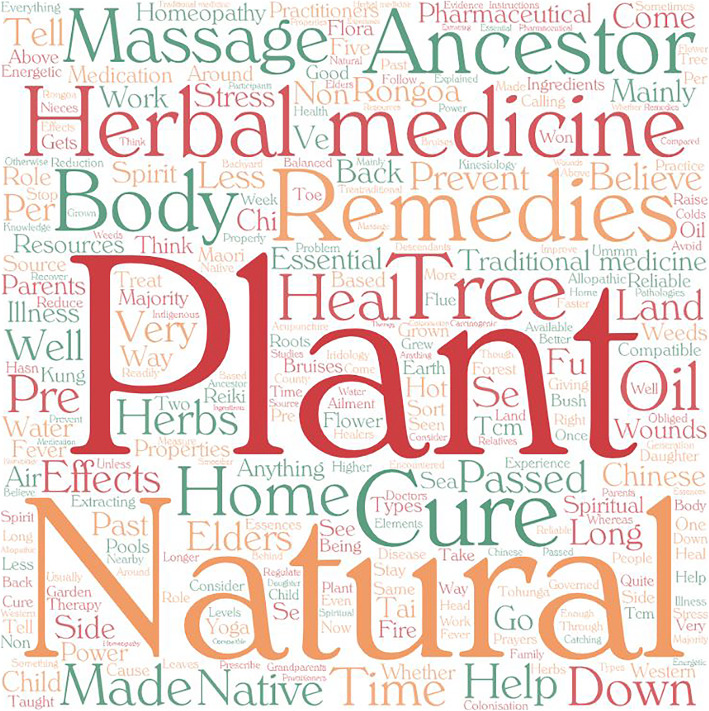
Word cloud created from the original quotes collected from the participants’ perspectives of traditional medicine. Font size correlates with the greater frequency of the word used in the interview

All participants had different understanding of TM and had described them differently, but plants and herbal medicine were discussed consistently (Fig. 1).

*“To me TM is all about non-pharmaceutical medicine, it’s about natural source of remedies and use any plant-based medicine….TM is mainly like extracting from the plant and use it” P13.*

*“Chinese herbs” P1.*

*“Herbal medicine comes from plants” p5.*

*“Herbs/weeds grown from home for home use” P6.*

*My experience of TM is traditional Chinese medicine, homeopathy, massage therapy, kinesiology, energetic medicine, herbal remedies, flower essences, essential oil remedies. I consider TM to be all of the above, anything other than allopathic medicine” P7.*

*“TM is the use of natural resources, native plants, and traditional remedies.” P9.*

*“TMs are not medication per se they are herbal types of medicines that we use for remedies” P12.*

Some participants thought that TM were made by their ancestors.

*“Made by my ancestors/rongoā practitioners, was made pre-pharmaceutical medications” P8.*

*“TM was used by our ancestors – pre-colonisation, it was the role of Tohunga to practise or use medicinal properties from native flora to heal any wounds or cure illness” P9.*

*“Anything and everything that our ancestors use or made (all-natural ingredients) to help treat and prevent any ailment or disease. Just what my grandparents and parents have used or taught me.” P11.*

Other definitions of TM were discussed such as:

*“Tai Chi Kung Fu” P1.*

*“Massaging is good and sometimes we like to go to hot pools, it also helps with well-being and reduce stress” P2.*

*“Rongoā Maori, Acupuncture, homeopathy iridology, massage, essential oil, yoga, reiki, forest and garden time – Stress reduction” P6.*

#### Sub-theme 1b: involvement of spirituality

Some participants believed the healing process was contributed by higher power.

*“ummm, just through spiritual, and that’s why we use to help cure any pathologies that we encountered. But at the same time, it’s spiritual healing and passing down from the ancestor. That is something we just so used to it and it’s not explained to us properly”. P13.*

*“Our body is governed by the five elements fire, water, air, earth, and spirit, and if any of these is not balanced then it is likely to cause problem in the body” P1.*

*“There are healers who use some sort of prayers and calling spirits to help with healing” P12.*

The higher power may come from ancestors, giving power to their descendants.

*“My ancestors have used them and passed down to me…I’ve seen it work so I use it” P11.*

*“…you are with your elders; you are obliged to use it as it has been passed on from generation….” P12.*

#### Sub-theme 1c: involvement with the environment

There was a strong correlation between the environment and TM. Most people believed TM works because it came from Mother Nature.

*“Indigenous people had cures from land. Majority of the natural ingredients are from the land/sea” P8.*

*“Back at my home county, majority of herbs or plants with medicinal properties are readily available in the backyard or the nearby bush and trees.” P11.*

*“TMs are made from plants, leaves, the tree and the roots that grew from our land….they are very effective and are compatible with the body with less side effects…“P14.*

#### Sub-theme 1d: knowledge and usage of TM

The knowledge and usage of TM were passed down from generation to generation. The family elders were gifted with this knowledge and experience from their parents. They were usually the skilled ones that carry out these TM on the younger generations.

*“It has been passed down from the past, and because it worked in the past, so we use it” P4.*

*“The Chinese doctors prescribe TM, and I just follow their instructions” P5.*

*“I use any TM because I believe in prevention rather than cure” P7.*

*“Rongoā practitioners have knowledge about TM as they use natural resources, native plants and traditional remedies to heal any wounds or cure illness” P8.*

*“For the elders, they will be able to see the tree and tell whether it is the right tree, whereas for me, I won’t be able to tell whether the tree is use for TM or not” P12.*

*“We usually take our family/relatives all the way back home, and they will stay there for a week or two to get treatment from the elders” P13.*

TM was mainly used because it had been around longer than western medicines.

*“TM has been around for a long time and I think it is quite reliable. I think western medicine is not very reliable as it hasn’t been used long enough compared to TM” P3.*

Some participants use TM because they believed TM could be used to improve one’s health.

*“TM can regulate the body and we use it as a preventative measure” P4.*

*“I use it to raise levels of wellness e.g. to recover faster from or avoid catching colds/flue” P6.*

For some participants, they explained how their parents had used TM on them when they were young, so they carried on with this practice.

*“My parents used it on me and now I use it on my nieces” P11.*

*“I believe when a child gets a fever, it is likely that they may have some bruises. So, when my daughter gets fever, I will do some massage to smoother the bruises. So that’s what we do, we use oil and the water and massage the child from the head all the way down to the toe. Only did it once and my daughter got better” P14.*

The usage of TM was favoured over conventional drugs because of its extensive natural activity and advanced safety margin.

*“TM is more natural, and we have been using it for a long time” P1.*

“*…. they are very effective and is compatible with the body with less side effects” P14.*

*“Unless studies have said it is carcinogenic then I will stop, otherwise I will use it even though there is no evidence behind it”. P12.*

### Theme 2: current practice and experience with TM

#### Sub-theme 2a: TM practices in dental/non-dental pathologies in New Zealand

**Fig. 2:**
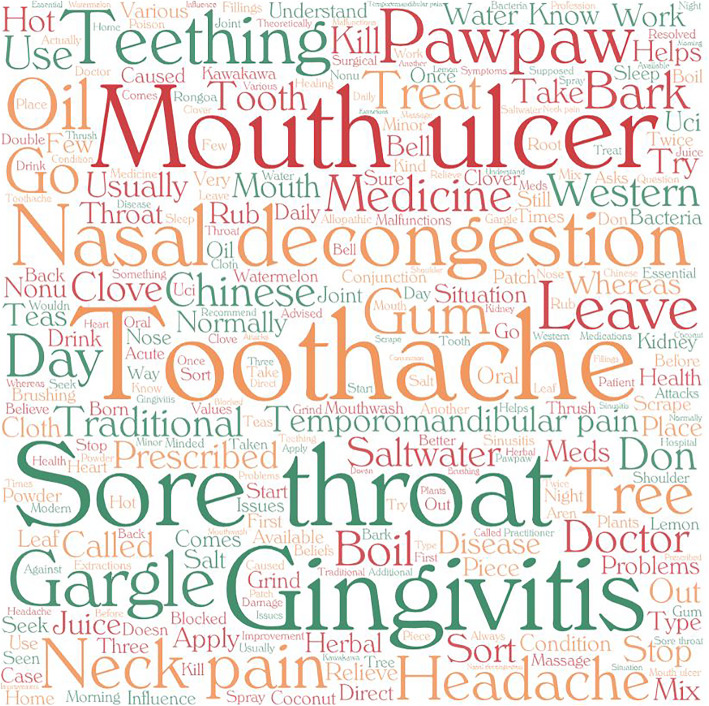
Word cloud created from the original quotes collected from the participants’ current practice and experience with traditional medicine. Font size correlates with the greater frequency of the word used in the interview

All these participants used a variety of TM for dental/non-dental pathologies that could be ranged from massage, praying to using plants (Fig. 2). There were also similarity and differences between the use of TM in different ethnic groups. A list of TMs used to treat certain head/neck/mouth conditions were listed below:

#### Toothache:

*“Clove oil for toothache” P7.*

*“Clove on sore tooth to relieve pain” P8.*

*“I understand there have been various bark teas available for toothache” P10.*

*“…you know the bark of the paw paw tree; we will scrape it and boil it with hot water and then gargle to kill off pain for toothache” P12.*

*“so we use the paw paw tree for toothache….normally use the paw paw bark….boil it and try get the juice out of the paw paw bark and gargle it but you don’t drink it” P13.*

#### Mouth ulcers:

*“Saltwater to gargle or apply salt on the ulcer. Use it for a few days and usually once or twice a day, normally before sleep” P1.*

*“I use a Chinese TM called watermelon powder for mouth ulcer.” P4.*

*“I use Kawakawa leaf three times daily for mouth ulcers” P6.*

*“For ulcers, there is a type of leave we use called the bell tree. They mix the leaves with water and use as a mouthwash and they use it for oral thrush too” P12.*

#### Gingivitis:

*“The saltwater can also be used for gum diseases as the gum diseases are usually caused by bacteria” P1.*

*“Coconut oil brushing for tooth and gum health” P7.*

*“Boil the leaves of the nonu tree and use the juice to gargle” P14.*

#### Nasal decongestion:

*“There is a Chinese herbal spray that I was prescribed by my doctor which I use for blocked nose” P5.*

*“For sinusitis or nasal decongestion, they will use lemon leaves and another sort of plants we used back home called Uci.” P12.*

#### Teething:

*“Clove leaves for treating teething, you grind clover leaves and place them in a piece of cloth and rub it on the gum for about 3 to 5 days.” P11.*

#### Sore throat:

*“If we have sore throat, we will gargle with saltwater” P2.*

#### Temporomandibular joint pain:

*“Massage for TMJ malfunctions, neck and shoulder issues which have a direct influence on the TMJ” P7.*

#### Headache:

“*I use Chinese essential oil for my headache” P4.*

#### Neck pain:

*“I have used a Chinese herbal patch that was prescribed by the Chinese doctor for neck pain and I apply it morning and night”. P3.*

#### Sub-theme 2b: Relationship between Traditional and Western Medicine.

Even though TM was widely used by the participants, there were some circumstances which they would still seek for western medicines. Majority of the participants considered TM as their first line of treatment and only sought for western medicines when the TM did not relief their symptoms.

*“There is some situation where TM is not helpful, e.g. tooth extractions, fillings, heart attacks” P7.*

*“We normally start with the TM for minor problems…if there are no improvement with TMs then we will seek western medicine” P1.*

*“I would use TM first, but if it doesn’t get better than I would go for western medicine” P3.*

*“When TM has not resolved the condition then I use allopathic treatments” P7.*

*“I still this day use TM because of it values and my beliefs” P11.*

*“It is something I was born with, so I actually believe in it. I’m like all for it. P12”.*

*“…. when it comes to surgical sort of treatment, I’d rather go for the hospital but when it comes to medicines I would go for TM.” P13.*

Some participants would consider using both methods.

*“I would use TM in conjunction with doctor prescribed medications” P8.*

*“Advised by a Rongoa practitioner not to stop the prescribed meds and use the TM in conjunction with the western meds” P8.*

However, there was one participant that would use TM only if she had seen it work.

“*I would use TM in situation where I have seen it work. If I am double minded and I am not very sure, then theoretically if I know it is not supposed to work that way then I wouldn’t use it” P1.*

Some believed that TM could cure the root of the problems.

*“TM treat the root, whereas western medicines treat the symptoms” P1.*

*“TM helps with healing whereas western medicines are used to treat acute conditions” P9.*

One participant thought that TM was safer than Western medicine.

*“Western medicine is like some kind of poison. They said it will damage your kidney, and if you have kidney problems, don’t take it, whereas this is not the case with TM” P2.*

When TM was discussed with western medical practitioners, they usually didn’t recommend patient taking TM.

*“I always question my doctor about TM, and he said because of the profession as a doctor of modern medicines, they don’t recommend patient to use or take TM but they aren’t against it and say it should be taken as an additional medicine” P11.*

However, most participants worked with their western health professionals when taking TM. If the western health professionals recommended against the use of TM, their advice was generally followed.

***“****I usually work with my doctor when taking TM…if he asks me to stop, then I will stop” P6.*

### Theme 3: barriers to accessing TM in New Zealand

#### Sub-theme 3a: lack of traditional practitioners

**Fig. 3:**
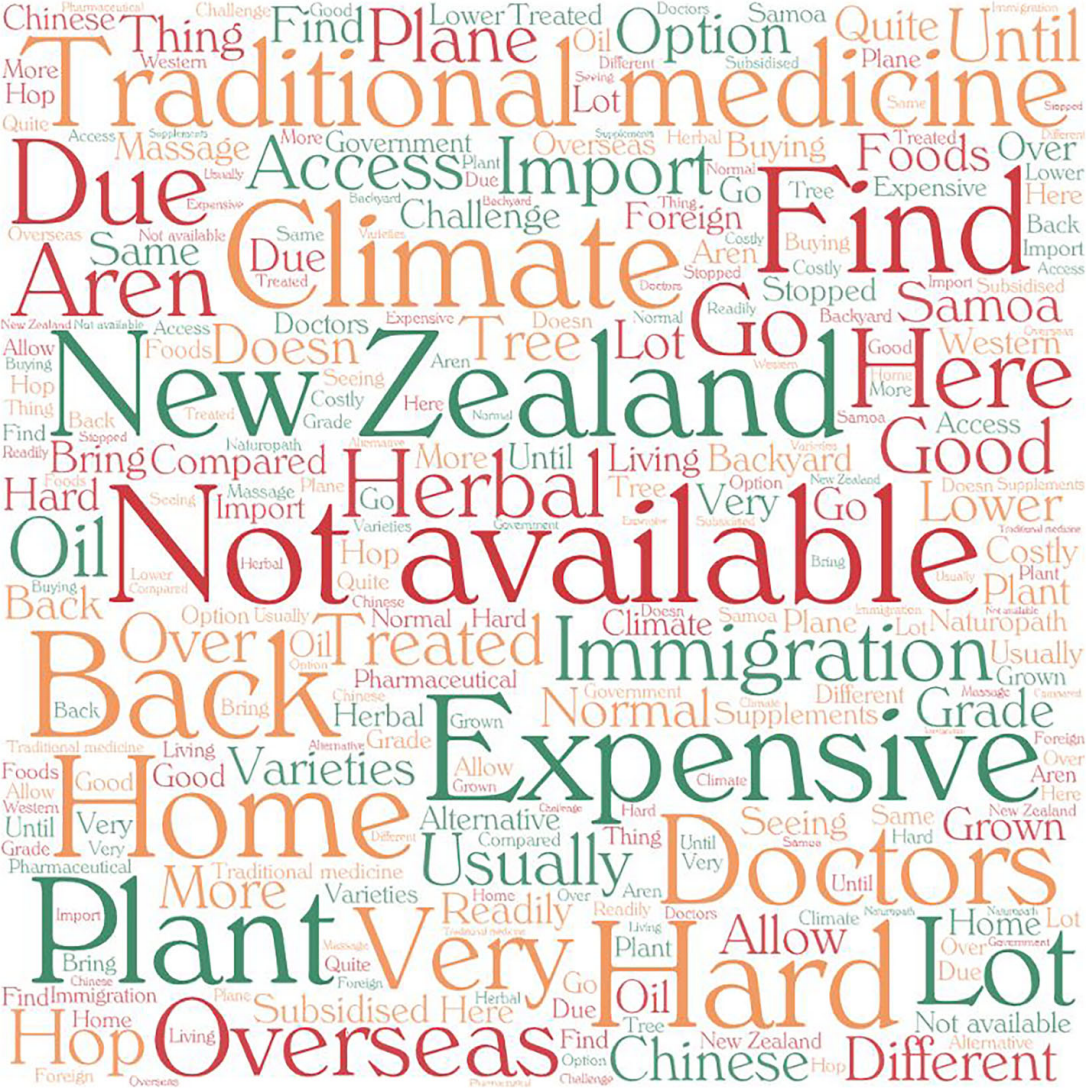
Word cloud created from the original quotes collected from the barriers to accessing traditional medicine in New Zealand. Font size correlates with the greater frequency of the word used in the interview

In New Zealand, experienced traditional practitioners were not readily available (Fig. 3). One would have to return to their country if one wanted to be treated by a traditional practitioner.

*“There are a lot of Chinese doctors in New Zealand, however, they aren’t as good as the doctors back home” P1.*

*“The only option to be treated with TM is to hop on the plane and go over to Samoa to be treated” P13.*

#### Sub-theme 3b: lack of traditional plants

Different climates and conditions as well as the immigration law had limited the sources of these herbal plants in New Zealand.

*“In New Zealand, it is very different, back home you can use TMs until you are normal as there are more varieties of TM compared to here…the herbal medicines are usually lower grade in New Zealand” P2.*

*“Immigration doesn’t allow plant/tree/foods into New Zealand, so it is hard to import herbal plants from overseas to New Zealand” P8.*

*“Access to TM in New Zealand is a challenge as it is hard to find the herbal plants…. back home, they are readily available in the backyard” P11.*

*“Due to the climate in New Zealand, some plants are not grown here…the only thing I could bring to New Zealand is the oil which I use it for massage” P12.*

*“Due to different climates, the plants in New Zealand are foreign to us and I can’t find the same plants I use back home” P14.*

#### Sub-theme 3c: cost of TM

Another aspect of limiting the use of TM in New Zealand was the cost.

*“It is not subsidised by the government, so it is quite expensive” P2.*

*“Seeing a naturopath and buying supplements are costly” P6.*

*“If the herbal medicines were available in New Zealand, they are usually very expensive” P11.*

#### Sub-theme 3d: adaptation and substitution

Since TM was not widely available in New Zealand, most people had opted for western medicines.

*“If I can find TMs then I would use it if I can’t find it then I will find alternative if not then I will use western medicines.” P3.*

*“The use of TM has stopped while living in New Zealand as it is hard to access…” P12.*

*“If I was back home I would use TM but since I am in New Zealand and have no access to TM, so I will have to use pharmaceutical medicines.” P13.*

## Discussion

This qualitative study investigated the perspectives, use of and access to TM by different ethnic groups in New Zealand. All participants shared their views on their use of TM. The reasons behind the use of TM were based on their experienced, culture and having witnessed the success of TM in the past. Three major themes and sub-themes were identified from our results, including the different perspective of each participant on TM, the current practice and experience of the participants, and barriers to accessing TM in New Zealand. This study had provided invaluable information about the perspectives, use of and access to TM by different ethnic groups in New Zealand.

### Traditional medicine perspectives

TM did not have a singular definition as shown in our results, and this was consistent with the literature [21]. Herbal and plant-based medicines, massage and spiritual healings were among some of the terms used to describe TM. TM can be divided into explicable and inexplicable forms. The explicable form of TM can be investigated, rationalised and explained scientifically such as the use of willow plant that contained the salicylates for fever and pains which led to the discovery of aspirin [22]. Most modern medications were developed through traditional medicine e.g. morphine, digoxin, quinine etc. [22]. Conversely, the inexplicable form of TM involves the spiritual, supernatural and magical that cannot be easily investigated, rationalised or explained scientifically [22]. TM was perceived to be a treatment that was natural, free of chemicals and harm. This was because most of the participants considered TM to have minimal side effects as they were harvested from plants. The knowledge and skills acquired by TM providers were through generational teaching from their families and different families with expertise in these areas [2]. Most participants justified their use of TM based on the family tradition and a long history of popular use. Some considered TM to be safe as it had been used for a long time. However, the crucial question underlying this statement was that should the absence of evidence of toxicity be taken as the safety of herbal medicine. Future studies in this area are required to support the use of herbal medicines.

Another important aspect that was discussed commonly was the spiritual component. The belief in the higher power having influence on health and the healing process was used in conjunction with other therapies. For example, the Rongoā Māori practises spiritual healing by controlling spirits and influence all aspects of life [23]. If a person was sick, the traditional healers would need to determine the cause of the imbalance before the illness could then be treated both spiritually and physically. In New Zealand, different ethnic groups utilised different types of TM. The use of TM was influenced by their context, background, where they were born and their age. Most participants would consider TM as their first line of treatment, and western practitioners were sought when there was no improvement with TM. Furthermore, the use of TM was affected by the lack of access to materials and the availability of the skilled providers. Therefore, some patients might choose to return to their home country for management and treatment. This study has provided valuable information about different ethnic groups perspectives on TM.

There are several TM educational institutions in New Zealand. They cover a large in-depth subject such as herbal *materia medica*, philosophy, principles and practice, safety, quality issues, and the medical sciences (anatomy, physiology, pathology, clinical skills, pathophysiology, phyto-pharmacy and herb-drug interactions). Their courses are normally 3 years full-time, and some of them can be registered with the New Zealand Association of Medical Herbalists, and the New Zealand Acupuncture Standards Authority.

### Use of TM in different conditions

3 medicines are still widely used by the participants based on their history, nature and time-tested scientific principles [27]. Integration of traditional practice into primary health care may aid in reducing oral health disparities and allow patients to discuss their use of TM with their health professional [28]. This allows dental practitioners to check for potential herbal – drug interactions before prescribing or initiating treatments. Our present study showed that the participants had been using a variety of TM in treating different conditions for dental care which was consistent with previous studies (Table 4) [2, 24–26, 29–41]. These conditions included gingivitis, toothache, teething, mouth ulcers, sore throat, temporomandibular joint pain, headache, neck pain and nasal decongestion. According to our findings, clove oil was used to treat toothache and teething. Clove oil contains eugenol and acetyl eugenol that provides an anti-inflammatory action and analgesic benefit [42]. Furthermore, Gupta reported that clove oil had used for hundreds of years by rural folks to prevent a toothache [27]. However, clove had been reported to exhibit anti-platelet activity and had potential interaction with warfarin [43]. Several traditional Chinese medicines also had interactions with warfarin [44]. In New Zealand, it was reported that over 60% of TM users did not realise that alternative medicines might interact with prescribed medications, and most of the users did not report to their health care professionals that they were using TM [45]. Hence, it was crucial for dental practitioners to screen for the use of TM, especially if patients were on warfarin therapy. By having awareness and knowledge of potential interactions of TM products with prescription medicines, it can improve the quality of the patient’s care. Although the safety profile of these TM is limited, the use of these TM would be continued, and TM would be considered as first choice in some of the patients due to the long history of use from the past. However, if studies showed TM was harmful, then our participants would choose not to use. The focus of this study is to explore the views, use of and access to TM among different ethnicities living in New Zealand, the impact of TM may have on dental care could be explored in future studies.

**Table 4:** Types of conditions treated with traditional medicine

Conditions	Current study	Previous studies
**Toothache**	Clove oil Bark teas Paw paw	Clove oil [2] Peppermint [2] *Acalypha* sp. Leave [18]
**Mouth ulcers**	Watermelon powder Salt water Kawakawa leaf Leaves from bell tree	Tree tea oil [2] Watermelon frost powder [19] Myrtle [20]
**Gingivitis**	Salt water Coconut oil Boil the leaves of nonu tree and gargle	Coconut oil [15] *Ocimum sanctum* 6% w/w [16] *Aloe vera* mouthwash [17]
**Nasal decongestion**	Chinese herbal spray Lemon leaves Uci plants	Moxibustion with Chinese herbal [24] Lemon juice [25] Acupuncture [26]
**Teething**	Clove leaves	None
**Sore throat**	Salt water	*Pawpaw* [18] Thyme [21]
**Temporomandibular joint pain**	Massage	Eucalyptus tree [18] Massage therapy [22] Acupuncture [23]
**Headache**	Chinese essential oil	Chinese herbal concoction [27] Hijama [28] Head banding [28]
**Neck pain**	Chinese herbal patch	Chinese herbal patches [29] Acupuncture [30] Acupressure [30] Cupping [30]

### Declining use of TM in New Zealand

The results from this study showed a possible decline in the use of TM in New Zealand. All participants had been exposed to TM at a young age and were confident and likely to continue with their use of TM. Most participants have lived in New Zealand for a long time, but still considered TM as their first line of treatment. The most important aspect that led to a decline in the use of TM was the cost. Even though some herbal ingredients were available in New Zealand, the cost associated with them were high as the supply of these ingredients were limited. Consequently, participants had to try other similar products or began to use western medicines. Although these participants practised TM, some of them feel there were limitations on the use of TM. Most of the participants indicated that they were comfortable using either TM and/or western medicine, depending on the condition that was being treated. If western medicine does not show any improvement in illness, some participants will choose to return to their countries to be treated by a skilled TM practitioner. Furthermore, by advertising local healers through social media or other networks can improve the awareness of such treatments being available in the neighbourhood and improve accessibility. To investigate the safe importation of TM ingredients could be explored. Since western medicines were subsidised by the government [46], the subsidisation of TM should also be addressed. With the limited access of TM in New Zealand, some participants in the study were concerned that TM might be lost in New Zealand. Further studies into the methods of improving accessibility and evaluating their effectiveness were therefore needed.

### The interplay of TM with Western medicine

Although the use of TM was questioned by some participants, the local health professionals were reluctant to accept the use of TM as most of them did not feel qualified to educate patients on this aspect [47]. It is important that physicians provide evidence-based information to patients and allow them to make informed decisions. However, the lack of evidence on the safety of TM had been a challenge for the physician to recommend the use of TM [48]. Participants were receptive to their western health practitioners if they were told not to use TM. In the present study, different patterns of TM use were identified. These included using TM as an alternative to western medicines, using TM with western medicine at the same time, and using TM prior to or after western medicine. The pattern of use depended on the confidence, the availability of healers and the accessibility to traditional plants [17]. In instances where TM was not available, then the participants would choose western medicines. However, if an illness did not respond to western medicines, then participants would try TM even if it meant to return to their countries to receive the treatment. This was because they had faith that it would work as they had seen or heard that it had worked on another person. It is crucial that western practitioners create an accepting atmosphere around TM use in their patients. This is vitally important because it allows patients to feel comfortable to disclose any TM use so that health practitioners may check for the possible contraindication and drug-drug interactions whilst respecting cultural views. It would be beneficial for dentists in New Zealand could have access to the education on the use of TM and relevant drug interactions to facilitate an open discussion with their patients who may choose to use TM.

## Strength and limitations

The respondents in this study may not be representative of the total population and all the ancient TM, however, this study provided a rich and deep description of the use of TM. Also, it broadened our understanding of the use of TM among different ethnic groups. This present study allowed participants to openly discuss their experience to uncover valuable meaning and to find a different type of objectivity which could be explored in further studies. The study had several strengths. Heterogeneous purposive sampling was implemented to ensure participants would provide in-depth answers and were of diverse ethnic background and age. All participants’ interviews were transcribed by the interviewer. By reducing interviewer bias, all participants were given the option to review their interview transcript and make any adjustment or further comments they wished as it gave participants the opportunity to review their response without the interviewer present. Participants who could only converse in Chinese had a Chinese translator to ensure they had a good understanding of the interview questions and could articulate their opinions with ease. Answers given in Chinese were translated by the Chinese translator as well as the interviewer – who spoke fluent Chinese – to minimise interpretation errors.

## Conclusion

Without doubt, the perspectives, use of and access to TM from different ethnic groups were addressed. This provided dental practitioners with an insight into the different sort of TM used by the population as well as the challenges faced by the majority of the participants in accessing TM. By understanding and acknowledging the use of TM, dental practitioners could create a supportive environment for patients to disclose their use of TM and as a result, improve patient care. Despite the participants’ effort in finding TM in New Zealand, more research is needed to investigate the effectiveness of these TMs and whether they could play a role in improving oral health.

## Data Availability

The datasets used and/or analysed during the current study are available from the corresponding author on reasonable request.

## Abbreviations

TM: Traditional medicine
